# Supplementary material for: Dengue virus co-opts innate type 2 pathways to escape early control of viral replication
Source: Commun Biol. 2022 Jul 22;5:735. doi: 10.1038/s42003-022-03682-5 (PMC9306424; doi:10.1038/s42003-022-03682-5)
Supplement: Supplementary file 2 — Supplementary information [file 42003_2022_3682_MOESM2_ESM.pdf]

Dengue virus co-opts innate type 2 pathways to escape early control of viral replication

Supplementary Information

Fonseka CL[1,2], Hardman CS[1], Woo J[1,3], Singh R[1], Nahler J[1], Yang J[1], Chen Y-L[1], Kamaladasa A[4], Silva T[4,5], Salimi M[1], Gray N[1,3], Dong T[1,6], Malavige GN* [4], Ogg, GS*[1,6]

[1]MRC Human Immunology Unit, MRC Weatherall Institute of Molecular Medicine, University of Oxford, Oxford, United Kingdom

[2]Department of Medicine, Faculty of Medicine, University of Ruhuna, Galle, Sri Lanka

[3] MRC WIMM Centre for Computational Biology, Medical Research Council (MRC) Weatherall Institute of Molecular Medicine, University of Oxford, Oxford, United Kingdom

[4]Allergy Immunology and Cell Biology Unit, Department of Immunology and Molecular Medicine, University of Sri Jayewardenepura, Nugegoda, Sri Lanka

[5] General Sir John Kotelawala Defence University, Rathmalana, Sri Lanka

[6] Chinese Academy of Medical Science (CAMS) Oxford Institute (COI), University of Oxford, Oxford, United Kingdom.

*Contributed equally

Correspondence - Graham.ogg@ndm.ox.ac.uk

This PDF includes:

Supplementary figure 1: Staining for blood ILC2 in healthy, DF and DHF samples.

Supplementary figure 2: ILC2 infection and type 2 cytokine production.

Supplementary figure 3: Receptors facilitating dengue virus attachment in ILC2.

Supplementary figure 4: Th2 and Tc2 cell sorting.

Supplementary figure 5: Dengue infection of pan-T cell, Th2 and Tc2.

Supplementary figure 6: Exogenous IFN-β controls dengue viral infection in ILC2.

Supplementary figure 7: Effect of ILC2 infection with IL-4 and IL-13 inhibition.

Supplementary figure 8: Urinary PGE_2 metabolite PGE-M levels in patients with acute dengue.

Supplementary figure 9: Western blot image.

Supplementary table 1: Reagents used for stimulation and blocking experiments

Supplementary figures

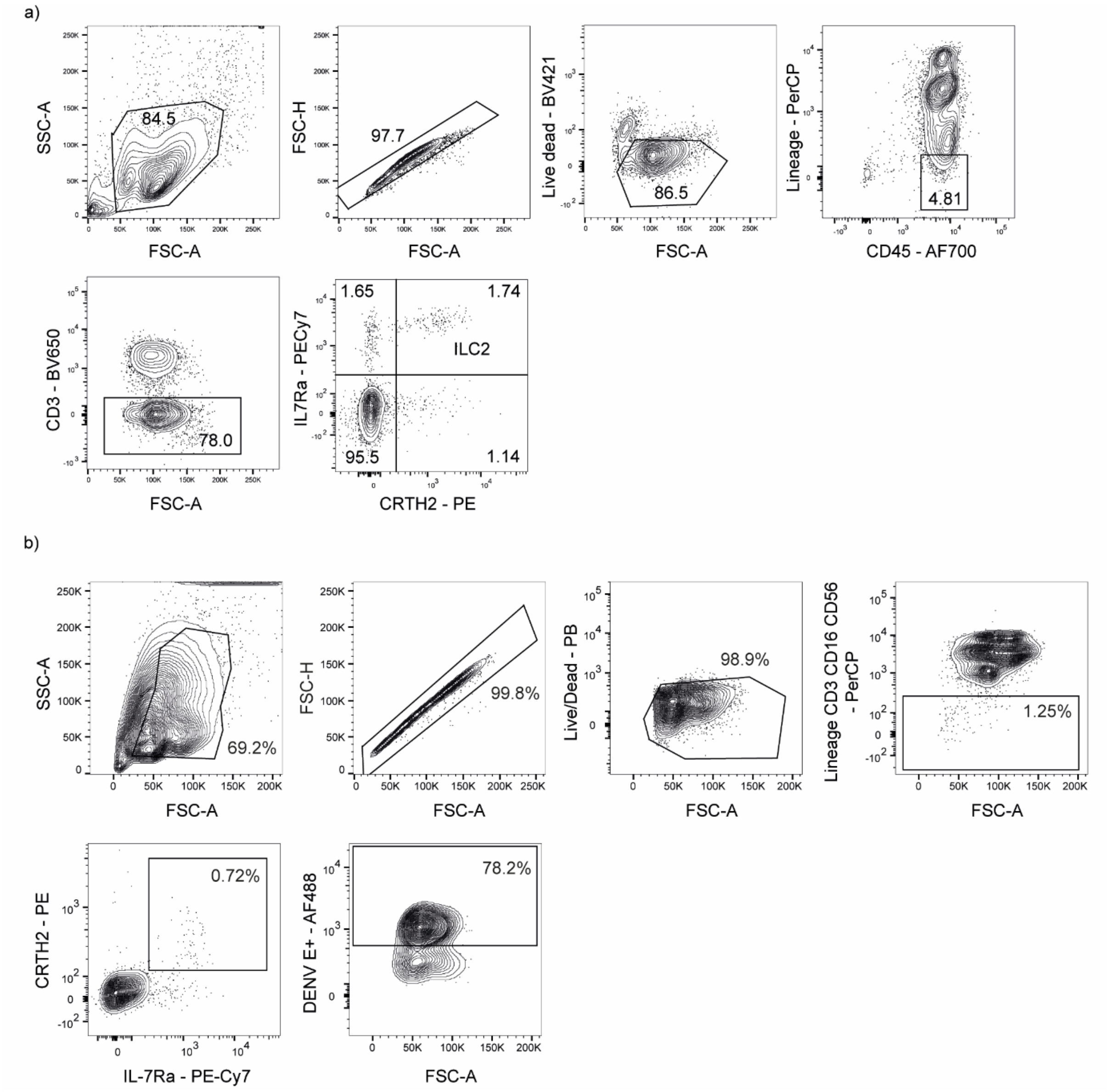

Supplementary Figure 1: Staining for blood ILC2 in healthy, DF and DHF samples. (**a**) Gating strategy for isolation of ILC2 of three clinical groups: Healthy individuals (HC), dengue fever (DF), dengue haemorrhagic fever (DHF) for *ex vivo* RNA sequencing. ILC2 are CD45+/ lineage-/CD3-/CD16 & CD56-/CRTH2+/IL-7Rα+. (**b**) Representative flow cytometry plot of *ex vivo* staining of ILC2 in patient with dengue infection. Cryopreserved samples were thawed and stained for ILC2 (Live/CD45+/lineage-/CD3-/CD16-/CD56-/CRTH2+/IL-7Rα+) and intracellular dengue virus envelope protein.

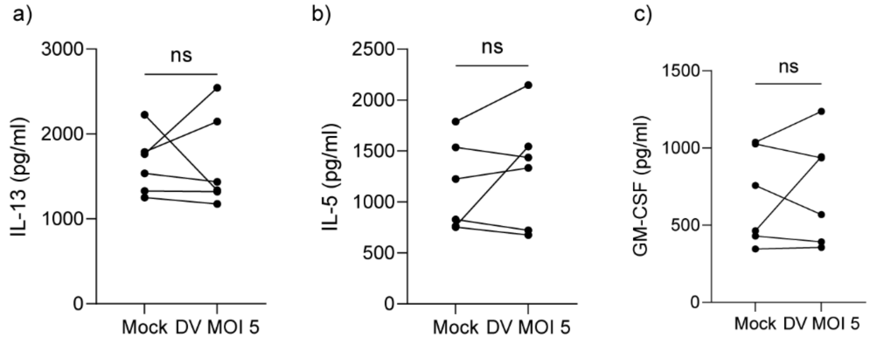

Supplementary Figure 2: ILC2 infection and type 2 cytokine production. Unstimulated blood ILC2 were infected with DENV MOI 5. Cells were washed and infectivity was examined after 48 hours. Supernatants of mock and infected ILC2 were collected and ELISA assays was performed on (**a**) IL-13, (**b**) IL-5 and (**c**) GM-CSF (n=5). Statistical significance was tested using T test. n.s. not significant.

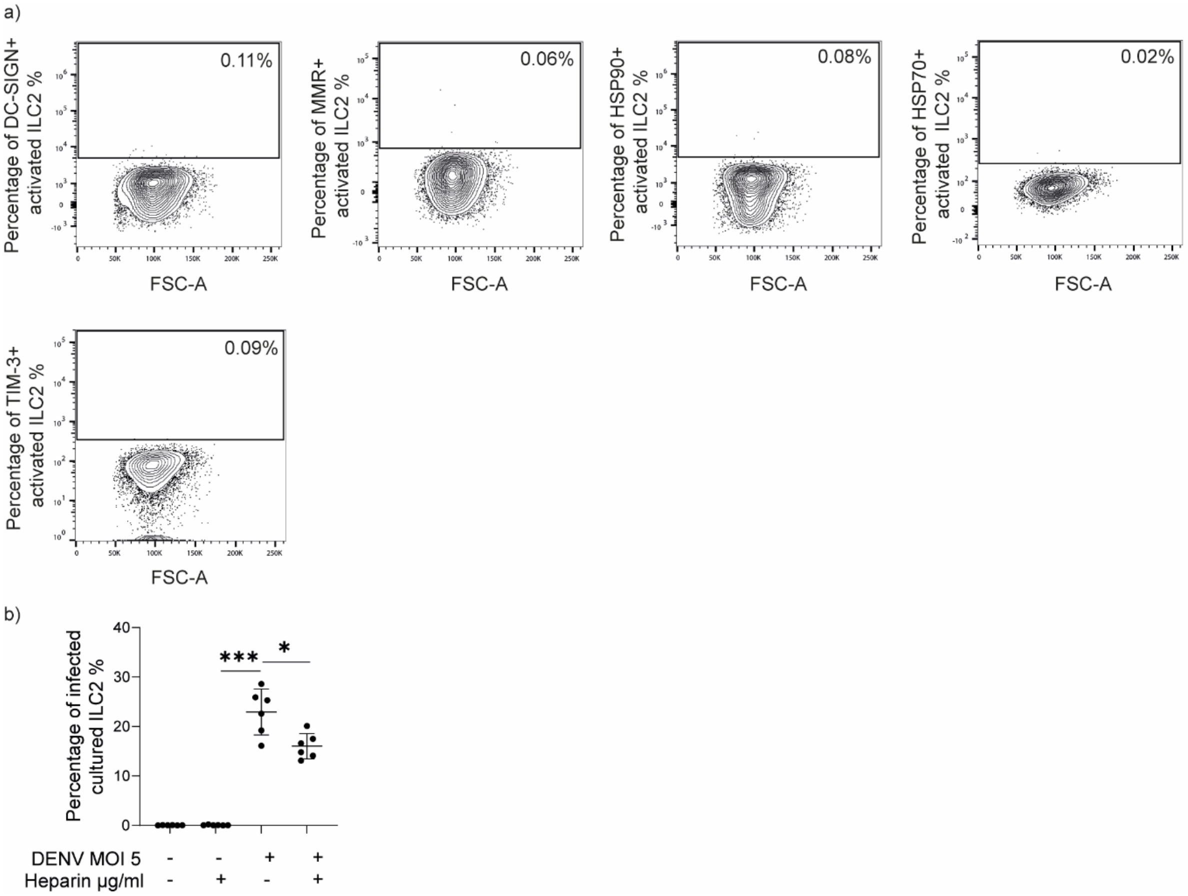

Supplementary Figure 3: Receptors facilitating dengue virus attachment in ILC2. (**a**) *Ex vivo* sorted and expanded ILC2 from healthy controls were activated with PGD_2 (200nM) for 24 hours and DC-SIGN, MMR, HSP90, HSP70, TIM-3 surface receptors were stained (representative figure from 3 donors). (**b**) *Ex vivo* sorted and expanded ILC2 from healthy controls were incubated with Heparin (Stemcell #07980) 100µg/ml concentration and incubated for 1 hour before infection. After 2 hours of DENV inoculation (MOI 5) cells were washed twice and plated in ILC2 media containing IL-2. Viral infection was assessed after 48 hours by staining for intracellular DENV E protein. Statistical significance was tested using one-way ANOVA with Tukey's multiple comparison test, data representative of 2 independent experiments (n=6). P *<0.05, *** <0.001, n.s. not significant. All error bars represent mean±SD.

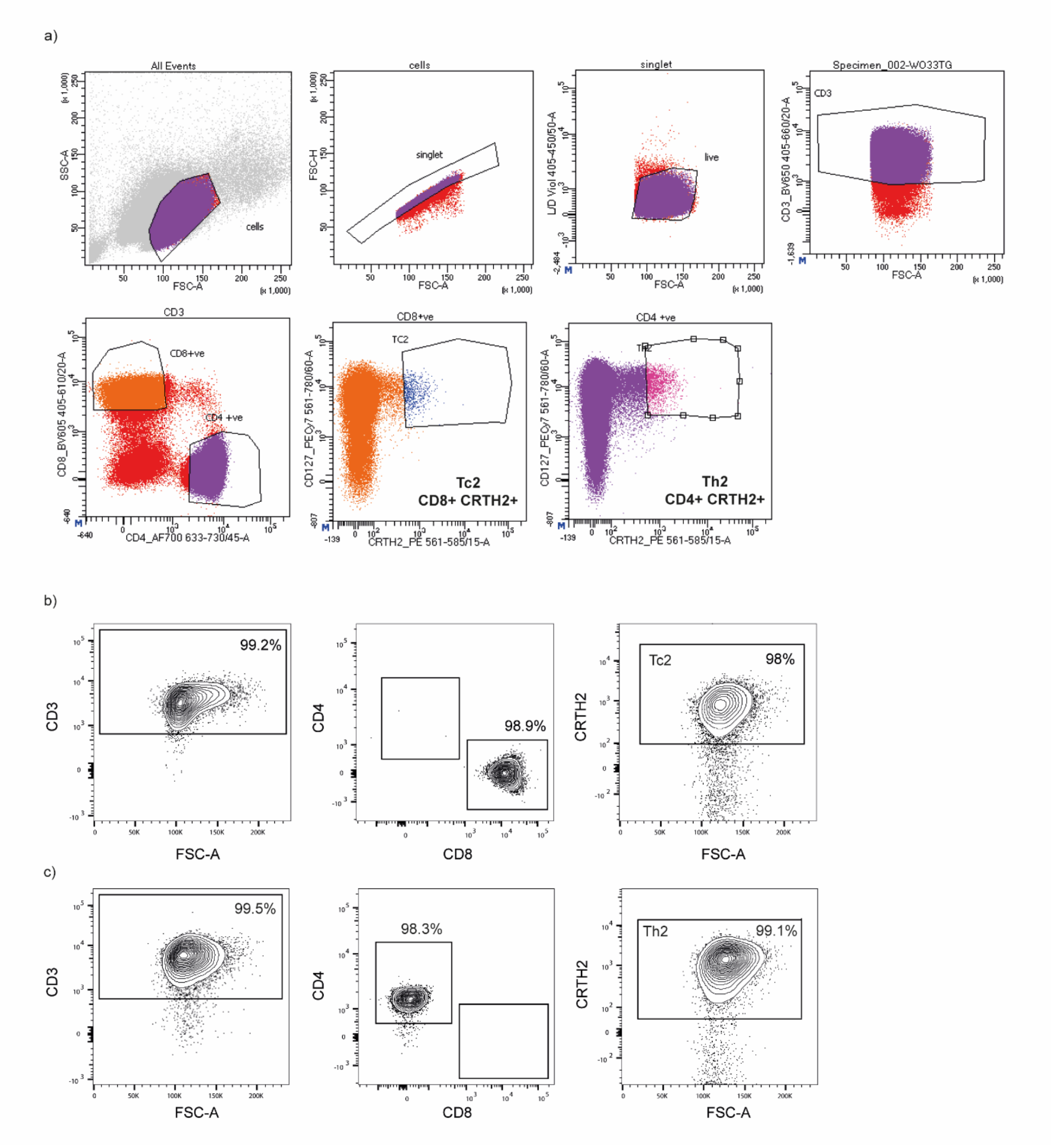

Supplementary Figure 4: Th2 and Tc2 cell sorting. (**a**) Th2 (CD3+ CD4+ CD127+ CRTH2+) and Tc2 (CD3+ CD8+ CD127+ CRTH2+) cells were sorted from healthy peripheral blood mononuclear cells. (**b**) The purity of Tc2 cells (CD3+ CD8+ CRTH2+) and (**c**) Th2 cells (CD3+ CD4+ CRTH2+) was confirmed by flow cytometry following in vitro expansion for 4-6 weeks.

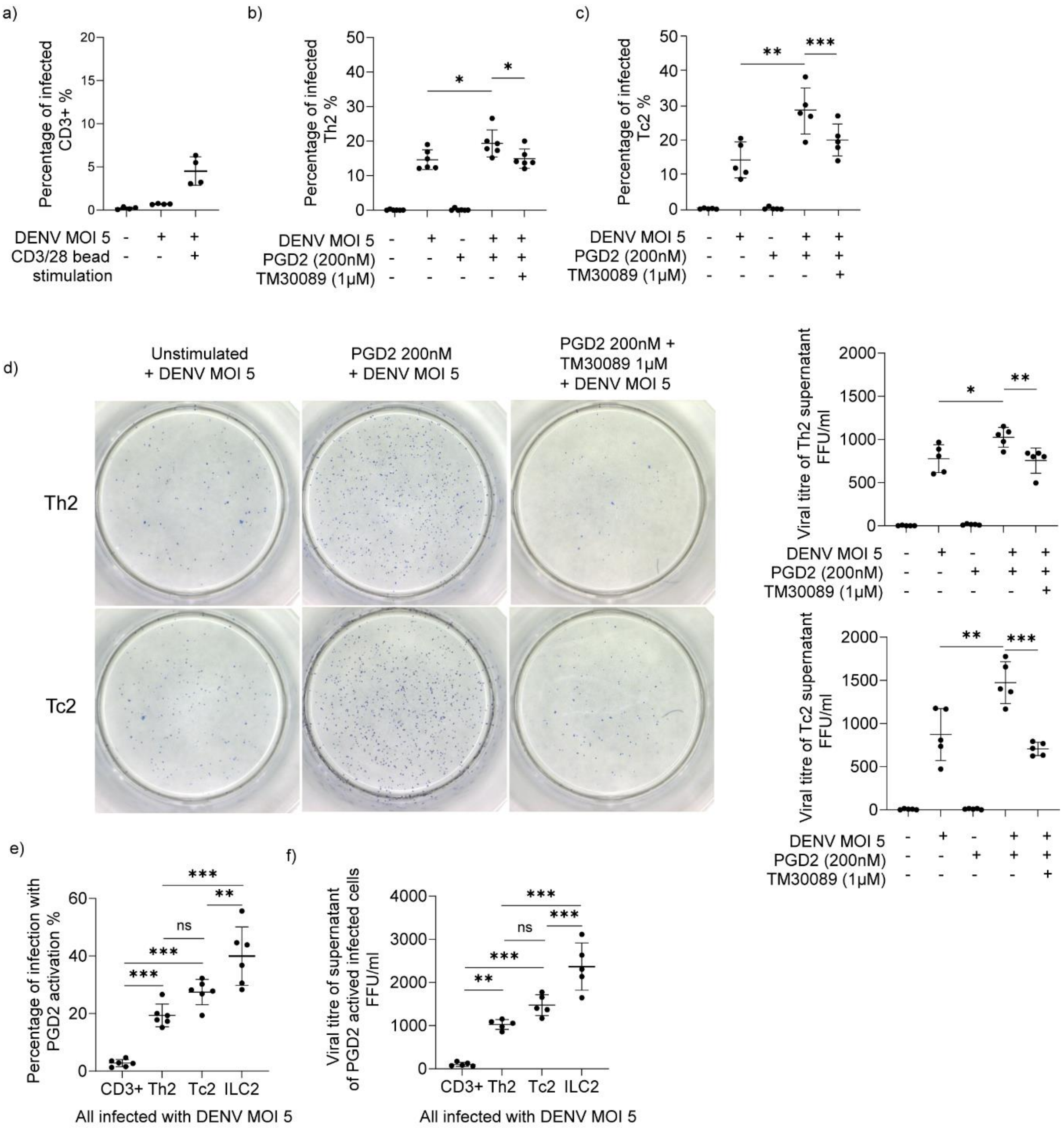

Supplementary Figure 5: Dengue infection of pan-T cell, Th2 and Tc2. (**a**) CD3+ MACS separated polyclonal T cells (pan-T cells) from healthy individuals were infected with DENV2 infection (MOI 5), and infection was assessed after 48 hours of incubation in T cell media with IL-2. For CD3/28 bead stimulation 1:1 bead to cell ratio was used and activated for 24 hours prior to infection. Statistical significance was tested using one-way ANOVA with Tukey's multiple comparison test, data representative of 3 independent experiments (n=4). (**b**) Th2 cells (CD4+ CRTH2+) from healthy individuals were sorted and expanded over 4 weeks. Unstimulated and PGD_2 (200nM) activated (for 24 hours) Th2 were infected with DENV2

infection (MOI 5), and infection was assessed. In the blocking condition, Th2 were treated with CRTH2 antagonist (TM30089 – 1µM) for 1 hour before treatment with PGD_2 (200nM). Statistical significance was tested using one-way ANOVA with Tukey's multiple comparison test, data representative of 3 independent experiments (n=6). P *<0.05. (**c**) Tc2 cells (CD8+ CRTH2+) from healthy individuals were sorted and expanded over 4 weeks. Unstimulated and PGD_2 (200nM) activated (for 24 hours) Tc2 were infected with DENV2 infection (MOI 5), and infection was assessed. In the blocking condition, Tc2 were treated with CRTH2 antagonist (TM30089 – 1µM) for 1 hour before treatment with PGD_2 (200nM). Statistical significance was tested using one-way ANOVA with Tukey's multiple comparison test, data representative of 3 independent experiments (n=5). P ** <0.01, *** <0.001. (**d**) 48 hours after infection, supernatants from mock and infected (PGD2 treated and PGD2 + TM30089 treated) Th2 and Tc2 cells were collected and added on Vero cells and then FFA assay was performed. Each focus is representative of an infected Vero cell. (n=5). P *<0.05, ** <0.01, *** <0.001. (**e**) CD3+ pan-T, Th2 and Tc2 from same healthy individual was incubated with PGD_2 (200nM) for 24 hours and were infected with DENV2 infection (MOI 5), and infection was assessed after 48 hours. Statistical significance was tested using one-way ANOVA with Tukey's multiple comparison test, data representative of 2 independent experiments (n=6). P ** <0.01, *** <0.001. (**f**) 48 hours after infection supernatants from PGD2 treated infected CD3+ pan-T, Th2 and Tc2 (DENV – MOI 5) were collected and added on Vero cells and then FFA assay was performed. Statistical significance was tested using one-way ANOVA with Tukey's multiple comparison test, data representative of 2 independent experiments (n=5). P ** <0.01, *** <0.001, n.s. not significant. All error bars represent mean±SD.

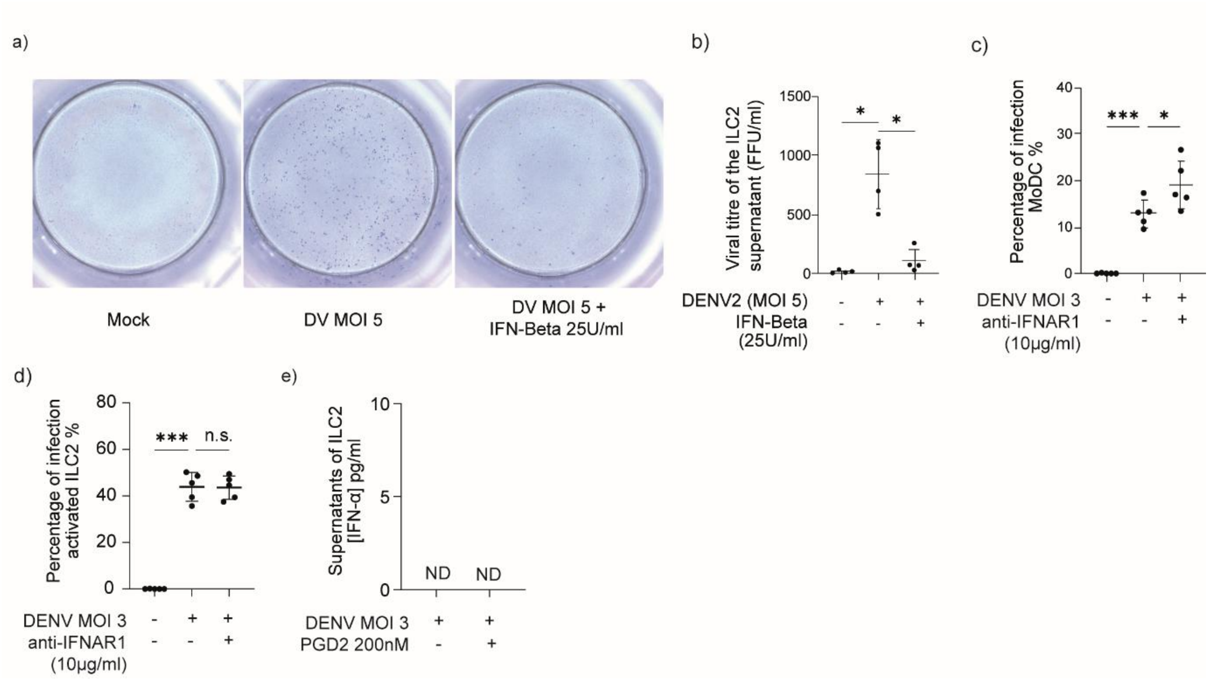

Supplementary Figure 6: Exogenous IFN-β controls dengue viral infection in ILC2. (**a**) Unstimulated and 24-hour IFN-β (250U/ml) treated ILC2 were infected with DENV (with MOI 5), washed and plated. Supernatants were harvested after 48 hours were added on Vero cells and Foci forming assay was performed. Each focus is representative of an infected Vero cell where intra-cellular DENV E protein was stained. A representative figure from 5 donors. (**b**) Viral titres ILC2 supernatants in unstimulated and IFN-β treated ILC2 were represented in multiple donors. (n=4, data representative of 2 independent experiments). Statistical significance was obtained using one-way ANOVA with Tukey's multiple comparison test, data representative of 2 independent experiments. P *<0.05. (**c**) CD14 MACS separated monocytes were differentiated with GM-CSF (100ng/ml) and IL-4 (10ng/ml) for 4 days and were infected with DENV2 (MOI 3) for 2 hours and the excess virus were washed. The cells were plated in GM-CSF and IL-4 for 48 hours. In blocking conditions anti-IFNAR1 (10µg/ml) added after wash step and incubated for 48 hours. Subsequently, intracellular DENV envelope protein was stained. Statistical significance was obtained using one-way ANOVA with Tukey's multiple comparison test, data representative of 2 independent experiments (n=5). P *<0.05, ***<0.001, n.s. not significant. (**d**) Activated ILC2 (activated with PGD_2 for 24 hours) were infected MOI 3 and incubated in ILC2 media containing IL-2 for 48 hours. In blocking conditions anti-IFNAR1 (10µg/ml) added after wash step and incubated for 48 hours. DENV infection was assessed after 48 hours. Statistical significance was obtained using one-way ANOVA with Tukey's multiple comparison test, data representative of 2 independent experiments (n=5). P ***<0.001, n.s. not significant. (**e**) ELISA for IFN-α was performed on supernatants of infected

unstimulated and activated ILC2 (n=5). ND = Not detectable. Data representative of 2 independent experiments. All error bars represent mean±SD.

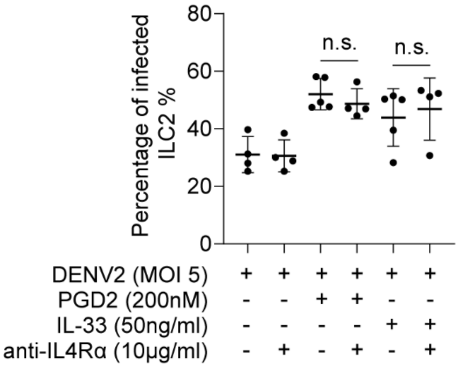

Supplementary Figure 7: Effect of ILC2 infection with IL-4 and IL-13 inhibition. ILC2 were treated with IL4Rα antagonist (10µg/ml) for 1 hour before treatment with PGD_2 (200nM) or IL-33 (50ng/ml) for 24 hours. ILC2 were infected with DENV2 infection (MOI 5), and infection was assessed after 48 hours. Statistical significance was obtained using one-way ANOVA with Tukey's multiple comparison test. (n=5, data representative of 2 independent experiments). n.s. not significant. Error bars represent mean±SD.

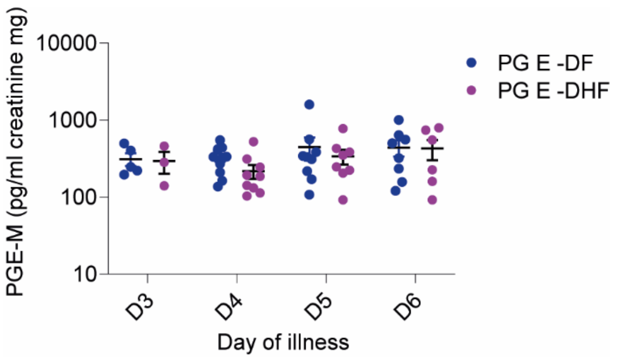

Supplementary Figure 8: Urinary PGE_2 metabolite PGE-M levels in patients with acute dengue. Urinary PGE-M were measured by quantitative ELISA, in those with DF (n = 12) and those who progressed to develop DHF (n = 10) in serially collected samples on 3rd, 4th, 5th and 6th days of illness. Error bars represent standard error of mean (SEM) and mean.

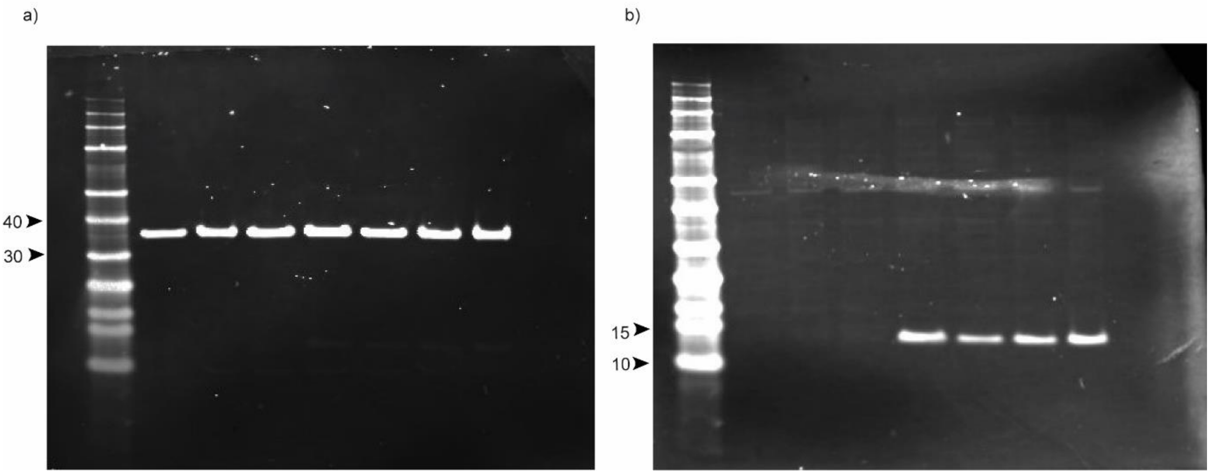

Supplementary figure 9: Western blot image. Uncropped western blot image of (a) GAPDH and (b) IFITM3.

Supplementary table 1: Reagents used for stimulation and blocking experiments

Reagent	Target	Source	Concentration
IL-33	ST2	Biolegend	50ng/ml
IL-25	IL-25R	Biolegend	50ng/ml
PGD_2	CRTH2	Enzo Life Science	200nM
LTE_4	CysLT1	Cayman Chemicals -20410	100nM
IFN-β	IFNAR1, IFNAR2	R&D Systems - 8499-IF-010	25-250U/ml
Anti-IL-4 receptor α	IL-4Rα	R&D Systems - AB-230-NA	10µg/ml
Anti-GM-CSF	GM-CSF	Mabtech - 23B6 - 3480-5N-500	5µg/ml
Anti-IFNAR1	IFNAR1	MMHAR-2	10 µg/ml
