# Supplementary material for: Dengue virus co-opts innate type 2 pathways to escape early control of viral replication
Source: Commun Biol. 2022 Jul 22;5:735. doi: 10.1038/s42003-022-03682-5 (PMC9306424; doi:10.1038/s42003-022-03682-5)
Supplement: Supplementary file 3 — Description of Additional Supplementary Files [file 42003_2022_3682_MOESM3_ESM.pdf]

Description of Additional Supplementary Files

File name: Supplementary Data 1
Description: Source data for the main figures in the paper.
