# Supplementary material for: Dengue virus co-opts innate type 2 pathways to escape early control of viral replication
Source: Commun Biol. 2022 Jul 22;5:735. doi: 10.1038/s42003-022-03682-5 (PMC9306424; doi:10.1038/s42003-022-03682-5)
Supplement: Supplementary file 4 — Supplementary Data 1 [file 42003_2022_3682_MOESM4_ESM.pdf]

Fgure 1C:

	HC1c	HC2b	HC2c	HC3b	HC3c	HC3	HC4c	DF2b	DF2c	DF2	DF3b	DF3c	DF3	DF4b	DF4	DHF1b	DHF1c	DHF1	DHF3b	DHF3c	DHF3	DHF4b	DHF4
CD38	0	0.166853683	0.217965	0.1673242	10.13746	0.64152	0.124822	74.20125	282.3693	58.00911	4.677036	1.352411	23.45087	230.5452	129.9976	76.44541	205.7182	149.3864	29.428	175.3114	52.11818	35.13023	56.62818
PTGDR2	317.542298	204.4090063	271.1009	272.02791	181.8508	59.93233	35.17528	48.87048	76.41927	96.02555	17.65753	180.4406	41.21463	31.41569	5.445858	212.1775	167.8657	153.4563	51.4365	86.21358	22.20146	118.136	138.8999
IL7R	651.034425	529.5203545	746.8675	535.05404	752.3203	420.2392	676.7903	133.3306	296.5441	193.8106	275.5874	386.4268	232.2026	126.5475	65.45685	507.2311	431.6548	541.0609	278.1389	448.5284	104.6979	287.3132	427.4074
IL2RA	68.02217	68.6153084	178.1057	113.99608	61.85704	50.2642	111.4572	10.62093	51.0024	21.92908	36.06333	111.9829	55.17974	16.87454	1.516212	123.0104	73.86298	121.6814	22.15842	91.18656	4.073994	34.03433	64.15853

Figure 1D:

HC	DF	DHF
0.31	1.34	1.42
0.14	0.37	6.27
0.19	1.55	2.28
0.1	1.09	0.41
0.24	0.65	0.58
0.29	1.33	0.4
0.46	2.44	0.22
0.5	0.21	0.56
0.53	0.04	0.69
0.14	0.57	0.57
0.41	0.31	0.12
0.55	0.6	0.25
0.48	0.78	0.81
0.48	0.76	1.04
0.77	1.4	0.33
	0.21	1.04
	0.14	
	0.23	
	0.3	
	0.02	
	0.17	
	0.47	
	0.2	
	0.59	
	0.23	
	0.47	
	0.16	
	0.7	
	0.28	

Figure 1F:

Healthy	Dengue patients
1.25	78.2
2.95	81.6
5.86	57.8
8.59	86.9
11.4	21.3
2.96	2.36
0.096	55.4
4.43	31.4
0.056	3.82

Figure 2A:

-	DV - HI	DVAb	DV	DV + DVAb
0.06	0.02	0.07	12	6.32
0.59	0.08	0.4	8.13	4.88
0.36	0.29	0.49	6.47	2.87
0.03	0	0	5.52	3.64
0	0	0.03	3.5	1.97

Figure 2B:

Mock	0.01	0.1	1	5
0.09	0.15	1.28	14.36	28.5
0.56	0.06	2.65	8.65	25.4
0.85	0.058	4.25	6.36	29.6
0.05	0.06	1.65	5.62	22.53

Figure 2C:

IL-33 (0-50ng/ml)

0	0.05	0.5	5	50
19.2	15.5	18.5	21.6	33.5
15.4	16.5	19.05	22.8	30.4
14	14.5	19.2	30	39.6
12.2	16.5	21.8	27.3	38.6

PGD2 (0-200nM)

0	0.2	2	20	200
19.2	20.2	22.3	31.1	51.4
15.4	20	20.7	34.8	48.8
14	18.1	21	45.4	63.5
13.8	16.3	24.2	46.2	66.1

LTE4 (0-100nM)

0	0.1	1	10	100
19.2	19.4	20.7	32.3	42.3
15.4	15.3	18.7	26.7	33.8
14	16.4	16	28.5	35.6
13.6	20.9	21.2	35.9	50.2

Figure 2E:

-	DV	IL-33 +DV	PGD2+DV	IL33 PGD2+DV
19.3	27.9	43.9	50.4	0.97
14.51	18.21	28.3	51.5	0.64
9.5	12.67	33.7	47.3	0.17
18.8	38.6	44.6	49.5	0.9
10.56	20.45	30.56	48.7	0.76

Figure 2F:

-	DV	IL-33 +DV	PGD2+DV	IL33 PGD2+DV
8	1064	1264	2532	2876
12	504	984	1744	2892
36	1104	2208	3312	4684
20	700	2364	2636	4052

Figure 3C:

	HC1c	HC2b	HC2c	HC3b	HC3c	HC3	HC4c	DF2b	DF2c	DF2	DF3b	DF3c	DF3	DF4b	DF4	DHF1b	DHF1c	DHF1	DHF3b	DHF3c	DHF3	DHF4b	DHF4
IFITM3	189.194647	735.1638201	704.9473	831.35225	453.2733	293.0615	815.7602	1600.563	1317.287	761.5987	3236.187	3108.786	4451.986	561.8489	467.1749	953.0102	735.6661	527.0203	461.421	508.8151	334.7859	2739.19	1551.522
ISG15	70.9026579	212.1717441	493.5787	447.8285	98.34552	289.1542	1989.077	1240.879	1230.304	931.7143	4778.48	3325.62	4008.094	846.8272	2026.233	1308.151	645.3055	499.2058	330.1089	412.9974	1145.761	943.2264	733.2953
OAS1	1.03824321	6.253867291	29.24711	21.887544	52.94976	2.725091	124.0728	98.60924	169.1883	51.95895	202.7952	207.2879	202.4456	96.26013	53.62771	200.6353	70.59182	49.21682	65.24099	53.00204	9.540101	168.1771	151.8644
RSAD2	1.72480814	1.487572795	0.138804	14.775603	16.52104	0.998631	36.8297	21.77595	47.38521	43.19275	156.7587	115.4441	155.7623	4.765667	22.56186	11.94294	14.97405	11.52799	7.797358	31.73825	1.929635	18.48007	27.29295
STAT1	32.3225071	56.18287154	40.84486	93.140064	61.13799	45.01127	59.85708	91.59174	100.0582	111.9754	219.2696	174.6735	219.528	70.9646	54.26206	33.12152	51.10727	77.6075	89.22053	93.86871	35.09446	62.53115	79.54369

Figure 3D:

IFITM3

Unstim	PGD2	IFN-Beta
0.184091942	0.18234742	2.236450789
0.05839455	0.04627613	1.40906811

OAS1

Unstim	PGD2	IFN-Beta
0.0146559	0.006985	1.472728
0.0152469	0.005718	0.950596

ISG15

Unstim	PGD2	IFN-Beta
0.416402	0.113959	8.441589
0.173831	0.094767	4.897649

RSAD2

Unstim	PGD2	IFN-Beta
0.581014	0.411995	2.312387
0.336111	0.267413	1.520265

STAT1

Unstim	PGD2	IFN-Beta
0.220123	0.172494	3.95088
0.282293	0.275931	4.143253

0.047929377	0.02854955	1.198031543
0.318585902	0.19303547	1.902644408
0.256435464	0.09654347	1.265435835

0.0100139	0.006379	1.102674
0.0134976	0.004846	1.307304
0.0125875	0.005765	1.265787

0.107034	0.036491	5.529766
0.298817	0.086242	10.14409
0.06324	0.028778	3.314778

0.29101	0.164306	1.726246
0.353828	0.258869	1.881046
0.23567	0.15154	1.305544

0.144545	0.101401	3.857092
0.354392	0.225384	5.109519
0.098722	0.066044	3.444555

Figure 3E:

-	DV	IFN-B 25IU/ml + DV	IFN-B 250IU/ml + DV
0	19.3	0.97	0.16
0.04	14.51	0.64	0.24
0.03	9.5	0.17	0.086
0.02	18.8	0.9	0.11
0.01	10.56	0.76	0.15

Figure 4A:

IFNLR1

Unstim	PGD2	IFN-Beta
0.017026189	0.00687898	0.038168355
0.024741068	0.01316431	0.049733984
0.016524664	0.00596457	0.071823115
0.009511281	0.00301757	0.034704535
0.035975805	0.00942917	0.0582151

Figure 4B:

OAS1

PGD2	IFN-β	PGD2 200nM + IFN-β	PGD2 20nM + IFN-β	PGD2 2nM + IFN-β
0.007218438	11.2320225	1.543234701	1.572002	4.2667674
0.009702309	6.58455326	2.254354769	2.434122	6.6077035
0.242781964	7.77930086	2.582510857	2.491872	5.138622

Figure 4E:

DV	DV + TM30089	PGD2 + DV	PGD2 + DV + TM30089	IL-33 + DV	IL-33 + DV + TM30089
12.3	10.8	47.4	16.7	50.1	23.8
14.6	11.2	49.2	22.6	50.3	24.5
16.1	12.4	47.6	35.4	51.4	42.8
16	12.4	58.1	27.9	39.5	23.3
27.3	20.1	57.8	17.8	28.2	17.8

Figure 4F:

DV	DV + MLK	LTE4 + DV	LTE4 + DV + MLK	PGD2 + DV	PGD2 + DV + MLK	IL33 + DV	IL-33 + DV + MLK
26.9	26.4	55.1	24.5	63.3	67.4	69.4	69.1
21.01	20.86	44.7	14.88	46.3	45.3	52.55	50.03
15.3	15.5	46.4	22.3	58.6	58.2	45.6	42.6
18.78	17.83	40.76	23.57	50.87	48.54	48.7	47.9

Figure 5C:

MMR%

-	U ILC2 sp	U ILC2 sp + IL4Rαb	U ILC2 sp + GM-CSFb	A ILC2 sp	A ILC2 sp + IL4Rαb	A ILC2 sp + GM-CSFb
15.9	29.9	28.3	20.5	68.9	67.6	52.4
	38.4	41.6	32.6	68.4	68	41.6
11.4	39.2	35.6	16.6	74.4	76	55.1
19.5	45.9	49.5	31.8	88.4	85.3	60.7
6.38	43.4	41	14.9	71.8	73.2	65.9
25.6	52.9	45.6	39.6	76.6	79.1	72.1

Figure 5D:

DV infection%

-	U ILC2 sp	U ILC2 sp + IL4Rαb	U ILC2 sp + GM-CSFb	A ILC2 sp	A ILC2 sp + IL4Rαb	A ILC2 sp + GM-CSFb
1.13	4.78	9.86	6.69	26.5	29.9	9.86
0.35	9.18	8.71	7.95	23.4	21.3	11
1.9	9.8	9.7	6.7	20	18.9	13.7
0.056	9.02	13.6	7.25	18.5	23.2	14.3
0.06	6.41	8.07	2.69	11.4	18.5	18

Figure 5E:

Mock	Mannan 20ug/ml	DV	Mannan 20ug/ml + DV
0.51	0.75	19.6	11
0.66	0.77	18.44	15.43
0.51	0.75	14.6	10.7
0.66	0.27	10.44	5.43
0.48	0.34	17.5	13.3

Figure 6A:

DF											DHF								
	603.14	633.58		81.55					130.41	105.48							331.23		409.67
983.36	167.07	1125.48	347.28	69.87	217.78	261.01	75.6	51.77	69.39	28.78	586.61	149.86	960.59	43.08	2271.23	262.53	201.65	59.99	247.55
236.71		517.81	131.59	72.19	457.01	155.14		172.52		34.11	177	371.43	49.68	467.13	3099.58	112.55	193.88	158.89	
493.45			39.19		57	194.7	88.12	67.56				1622.9	237.24	382.19	3073.02	114.74		20.79	

Figure 6B:

24 hrs before entering critical stage	Critical stage
149.86	371.43
	960.59
43.08	467.13
391.67	282.73
140.26	141.42
185.24	383.78
	458.85
